# True Lateral Eye Numbers for Extant Buthids: A New Discovery on an Old Character

**DOI:** 10.1371/journal.pone.0055125

**Published:** 2013-01-30

**Authors:** Xiaofeng Yang, Yusoff Norma-Rashid, Wilson R. Lourenço, Mingsheng Zhu

**Affiliations:** 1 College of Life Science, Hebei University, Baoding, Hebei Province, People’s Republic of China; 2 Institute of Biological Sciences, Faculty of Science, University of Malaya, Kuala Lumpur, Malaysia; 3 Department of Organismic and Evolutionary Biology, Harvard University, Cambridge, Massachusetts, United States of America; 4 Muséum National d’Histoire Naturelle, Département Systématique et Evolution, Paris, France; Lund University, Sweden

## Abstract

This study reports the results of a re-analysis of the number of lateral eyes in extant buthids. Specimens studied were confined mostly to those from China and the adjacent areas. 353 specimens belonging to 20 species (subspecies) and 8 genera were rechecked and found to have 5 pairs of lateral eyes contrary to earlier published works which reported the presence of 3 pairs of lateral eyes only. Combined with reported examples collected through reference investigation including 63 species from 16 genera, our study here shows a total of 79 species from 21 genera of scorpions spanning Asia and Africa had 5 pairs of lateral eyes. Reasons for not observing the presence of the extra lateral eyes are discussed and new protocols for examining specimens including using UV light are proposed to aid lateral eye recognition. Besides, a majority of genera in Buthidae are suggested to be in “Five-eye” model and a re-examination of all previously published taxonomic studies of buthid species is highly recommended.

## Introduction

Eye features are widely known to be important characters in the study of taxonomy in most arthropod groups. Scorpions have two types of eyes, namely, the median and lateral eyes. Most scorpions have two median eyes, only several species lacks. The number of lateral eyes varies among different major groups of extant scorpions. The evolution trend of this feature is unknown.

Koch [1] was the first to think highly of the number of lateral eyes in scorpion taxonomy. He attempted to classify the genera into families based on the number of lateral eyes: Scorpionidea (two pairs); Buthidea (three pairs); Centruridea (four pairs); and Androctonidea (five pairs). Lankester [2] pointed out that the number of lateral eyes was unstable and Koch’s classification was unreliable. Thus subsequent taxonomists did not use this character at higher taxonomic levels in extant scorpions. Later, Stockwell [3] further supported Lankester’s point and reported that Koch’s classification groups were inaccurate resulting in incorrect assignation of many of the genera. Kjellesvig-Waering [4] restudied fossil scorpions of the world and placed a high value on the disposition of the lateral eyes concluding that the reduction in lateral eye units was an evolutionary trend which led to the available recent taxa. Stockwell [3] affirmed Kjellesvig-Waering’s opinion in fossil scorpions in his unpublished PhD thesis, but he did not emphasize on the importance of lateral eyes in the extant groups. Soleglad and Fet [5] re-analyzed the higher taxonomic system with 105 characters selected from all extant families which included the lateral eyes counts. However they denoted the numbers from Buthidae and Pseudochactidae as “uninformative data”.

Huge subsequent taxonomic changes have taken place since the era of Koch. With a number of 971 species and 89 genera [6], family Buthidae is the largest family in Scorpionida, representing almost half of the species and genera of the known scorpions. All of the above suggest great potential significance and necessity on further research for family Buthidae on this subject. Hence the study on lateral eye counts in buthids is proposed in this current work.

## Materials and Methods

### Material Examined

A total of 353 specimens belonging to 20 species (subspecies) and 8 genera were reexamined including 18 species (subspecies) involved in 7 genera from China and adjacent areas of Asia, 2 species involved in 1 genus from Africa.

*Mesobuthus* (8 species and subspecies): *Mesobuthus martensii martensii* (Karsch, 1879), 55♂, 94♀, 30 juv, China: Liaoning, Hebei, Ningxia, Qinghai; *Mesobuthus eupeus mongolicus* (Birula, 1911), 4♂, 8♀, China: Inner Mongolia, Gansu, Ningxia, Xinjiang; *Mesobuthus eupeus thersites* (C. L. Koch, 1839), 10♂, 14♀, 2 juv, China, Uzbekistan, Kazakhstan; *Mesobuthus caucasicus intermedius* (Birula, 1897), 20♂,10♀, Kazakhstan, Uzbekistan; *Mesobuthus caucasicus przewalskii* (Birula, 1897), 1♂,1♀, China; *Mesobuthus karshius* Sun & Sun, 2011, holotype ♀, paratype 1♂,1♀, China; *Mesobuthus longichelus* Sun & Zhu, 2010, holotype ♀, 2 juv, China; *Mesobuthus bolensis* Sun, Zhu & Lourenço,2010, holotype ♂, paratype 1♂, China.

*Hottentotta* (2 species): *Hottentotta songi* (Lourenço, Qi & Zhu, 2005), paratype 1♂, 1♀, 1 juv, China; *Hottentotta tamulus* (Fabricius, 1798), 1♀, 1 juv, India.

*Isometrus* (4 species): *Isometrus* (*Isometrus*) *maculatus* (DeGeer, 1778), 2 juv, China; *Isometrus* (*Reddyanus*) *hainanensis* Lourenço, Qi & Zhu, 2005, Paratype 1♀,1♂, China; *Isometrus (Reddyanus) tibetanus* Lourenço & Zhu, 2008, holotype ♂. China; *Isometrus (Reddyanus) assamensis* Oates, 1888. 2♀,1♂, India.

*Lychas* (1 species): *Lychas mucronatus* (Fabricius, 1798), 29♂, 44♀, 4 juv, Vietnam, China: Yunnan, Hainan.

*Razianus* (1 species): *Razianus xinjianganus* Lourenço, Sun & Zhu, 2010, Holotype ♀, China.

*Sassanidotus* (1 species): *Sassanidotus gracilis* (Birula, 1900), 1♀, Iran.

*Kraepelinia*(1 species): *Kraepelinia palpator*, 1♀, Iran.

*Buthus* (2 species): *Buthus occidentalis* Lourenço, Sun & Zhu, 2009, 1♀, 1 juv, Mauritania; *Buthus draa* Lourenço & Slimani, 2004, topotype 1♀, Morocco.

### Examination, Photography, Measuration and Illustration

Specimens were examined and photographed using the white and violet lights (wavelength range: 390–420 nm). Photos of lateral eyes were captured using the equipment set-up model, Nikon SMZ1500 Stereomicroscope, Nikon CF Plan 10X Objective and Canon 60D SLR.

A standard histological technique was used to prepare longitudinal and transverse ultrathin sections of the lateral eyes. The technique involved preparation of specimen paraffin blocks which were sectioned using ultramicrotome with glass knives to obtain 60–90 nm sections. These sections were stained with hematoxylin, later examined, measured and photographed with Leica DM 2000 Microsystems.

Drawings were made by using Photoshop 8.01 and Wacom Intuos 3 pen tablet.

### Definition of Terms

**Figure 1:**
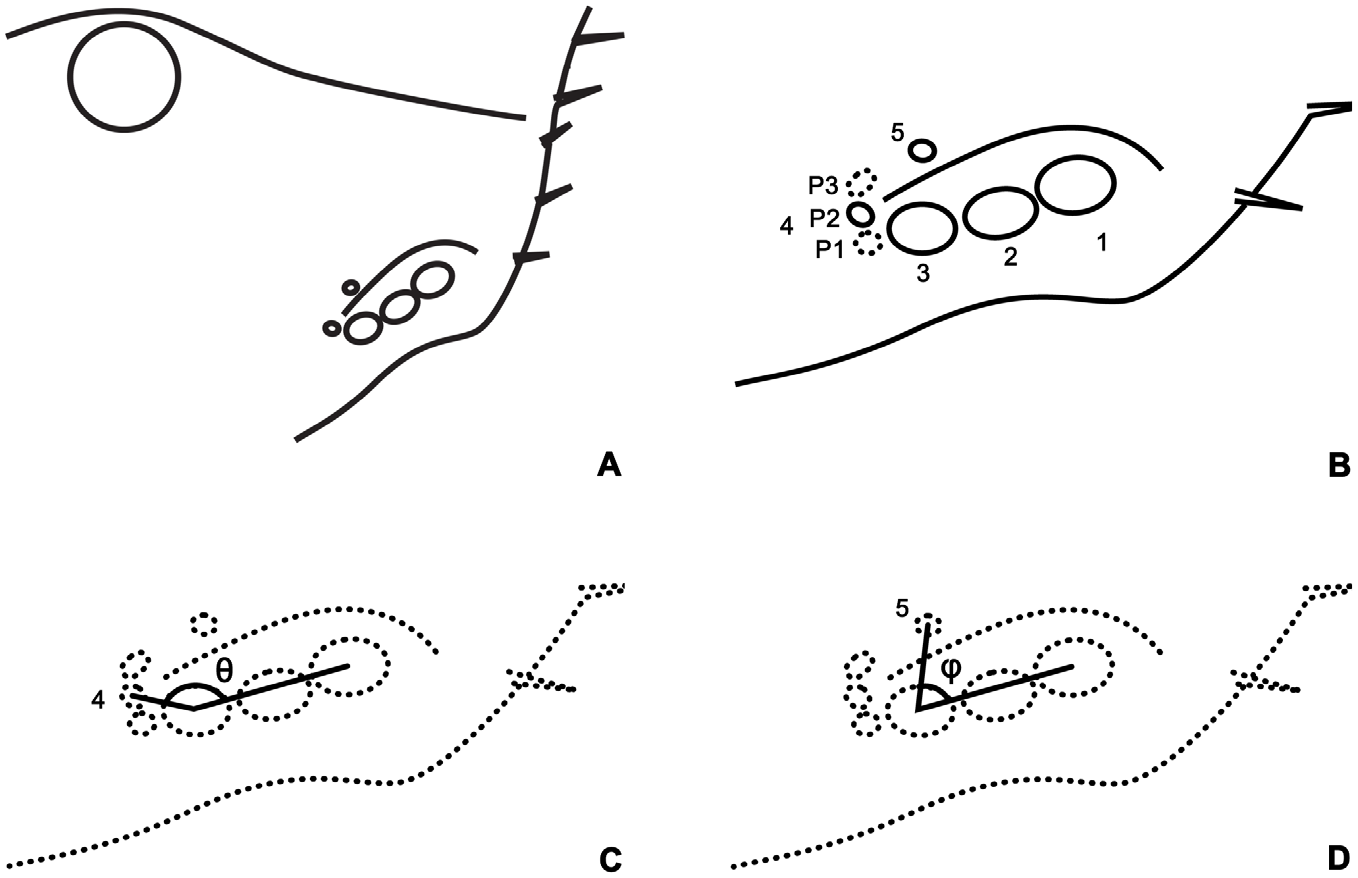
Models of lateral eye area. (A) The position of lateral eye area on Carapace; (B) Supposed model of 5 lateral eyes, P1, P2, P3 are 3 possible locations of the No. 4 lateral eyes; (C) Angle θ, indicating the location of No. 4 lateral eye; (D) Angle φ, indicating the location of No. 5 lateral eye.

There was no species found to have more than five pairs of lateral eyes and the relative position of each lateral eye was comparatively fixed within species (for details, see below). Therefore, five pairs of lateral eyes were suggested to be a standard model here and the ordinal number No. 1–5 refer to each pair of the lateral eyes for convenience of recording and describing (after Fleissner [7], [8]). The model described here is illustrated in Figure 1(A, B).

The location of the lateral eye No. 4 showed variation to a certain degree across species and sometimes, even within the same species. P1–P3 were assigned to show the three possible locations of the No. 4 lateral eye. Locations of No. 4 and No. 5 lateral eyes could also be determined by the angle formed by two lines, one through the center of the No. 4 (or No. 5) and No. 3, the other line through the center of the No. 1 and No. 3. Angle names θ and φ were assigned to use for describing No. 4 and No. 5 lateral eyes respectively. (Figure 1. C & D).

## Results

**Figure 2:**
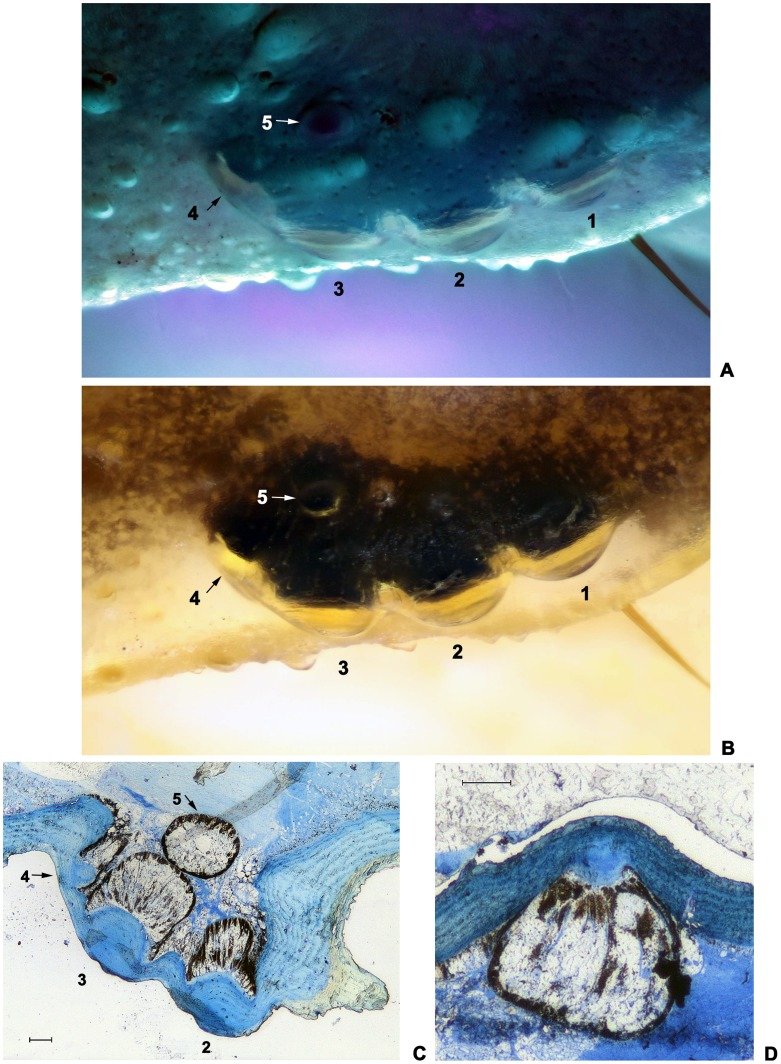
Lateral eyes of *M. martensii martensii.* (A) Under UV light; (B) Under white light; (C) Longitudinal section of No. 2–4 lateral eyes with transverse section of No. 5 lateral eye. 0.1 mm; (D) Longitudinal section of No. 5 lateral eye. 0.1 mm.

In the widespread Chinese subspecies, *M. martensii martensii*, we found 2 extra pairs of lateral eyes here labeled as No. 4 and No. 5 when examined under the UV light (Figure 2. A). In contrast, these 2 lateral eyes were difficult to recognize when specimens were examined under normal white light (Figure 2. B). We also found different depictions on this number in previous publications, i.e. 5 in Kishida [9], 3 in Qi, et al. [10] and Sun & Sun [11].

To confirm the existence of these extra pairs of lateral eyes and to discount confusion with tubercle structures on the carapace, we performed a dissection on the carapace to prepare histological sections for examination, which resulted in affirmation of eyes No. 4 and No. 5 (Figure 2. C, D). The measurements of the two micro-eyes were 0.12 mm in diameter in contrast to No. 1 to No. 3 eyes which were all 0.3 mm in diameter. Additionally, 55♂, 94♀ and 30 juv collected from 4 different locations were reexamined to avoid partial conclusion due to variation among local populations and individuals. The statistical result can be seen in Table 1 which shows good consistency with 5 lateral eyes.

**Table 1:** Status Update and Statistics of Rechecked Specimens.

Species/Subspecies	ReportedNumber	RecheckedResult	RecheckedSpecimens	Location(s)	Exceptions	ReferencesStudied
*M. martensii martensii*	3 & 5	5	55♂, 94♀, 30juv	China (Liaoning, Hebei,Ningxia, Qinghai)	5 specimens lack No. 4 on oneside; 1 specimen lacksNo. 2 on one side	[9], [10], [11]
*M. eupeus mongolcus*	3	5	4♂, 8♀	China (Inner Mongolia, Gansu,Ningxia, Xinjiang)	1 specimen has 6 lateraleyes on both sides	[11], [12], [13]
*M. eupeus thersites*	3	5	10♂, 14♀, 2juv	China, Uzbekistan,Kazakhstan		[12], [13], [14], [15]
*M. caucasicus* *przewalskii*	3	5	1♂,1♀	China		[11], [15], [16], [17]
*M. caucasicus* *intermedius*	3	5	20♂,10♀	Kazakhstan,Uzbekistan	1 specimen lacks No. 2;1 specimen has 6 lateraleyes on one side	[11], [15], [16], [17]
*M. bolensis*	3	5	1♂, 1♀	China		[18]
*M. longichelus*	3	5	1♀, 2juv	China		[16]
*M. karshius*	3	5	1♂, 2♀	China		[11], [18]
*H. songi*	3	5	1♂, 1♀, 1juv	China		[18], [19]
*H. tamulus*	5	5	1♀, 1juv	India		[20]
*I.* (*Isometrus*)*maculatus*	5	5	2 juv	China		[20]
*I.* (*Reddyanus*)*hainanensis*	3	5	1♂, 1♀	China		[21]
*I.* (*Reddyanus*)*tibetanus*	3	5	1♂	China		[22]
*I.* (*Reddyanus*)*assamensis*	3 & 5	5	1♂, 2♀	India		[20], [22]
*L. mucronatus*	5	5	29♂, 44♀, 4juv	Vietnam, China(Yunnan, Hainan)	1 specimen lacksNo. 5 on both sides	[20], [23]
*R. xinjianganus*	3	5	1♀	China		[24]
*S. gracilis*	3	5	1♀	Iran		[24]
*B. draa*	3	5	1♀	Morocco		[25]
*B. occidentalis*	4	5	1♀, 1juv	Mauritania		[25]
*K. palpator*	3	5	1♀	Iran		[26]

**Figure 3:**
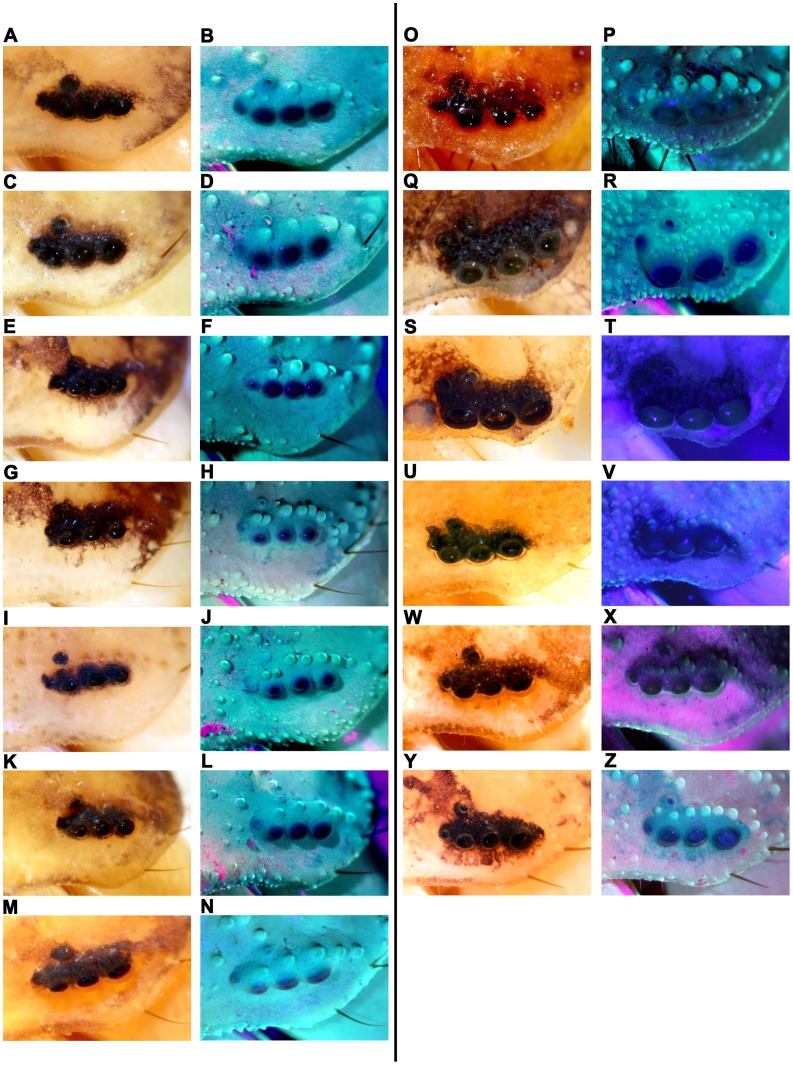
Lateral eye area of representive species and subspecies. (A)&(B) *M. eupeus thersites*, (C)&(D) *M. eupeus mongolcus*, (E)&(F) *M. caucasicus przewalskii*, (G)&(H) *M. caucasicus intermedius*, (I)&(J) *M. bolensis*, (K)&(L) *M. longichelus*, (M)&(N) *M. karshius*. (O)&(P) *H. songi*, (Q)&® *L. mucronatus*, (S)&(T) *I. (Reddyanus) assamensis*, (U)&(V) *R. xinjianganus*, (W)&(X) *S. gracilis*, (Y)&(Z) *B. draa*.

**Figure 4:**
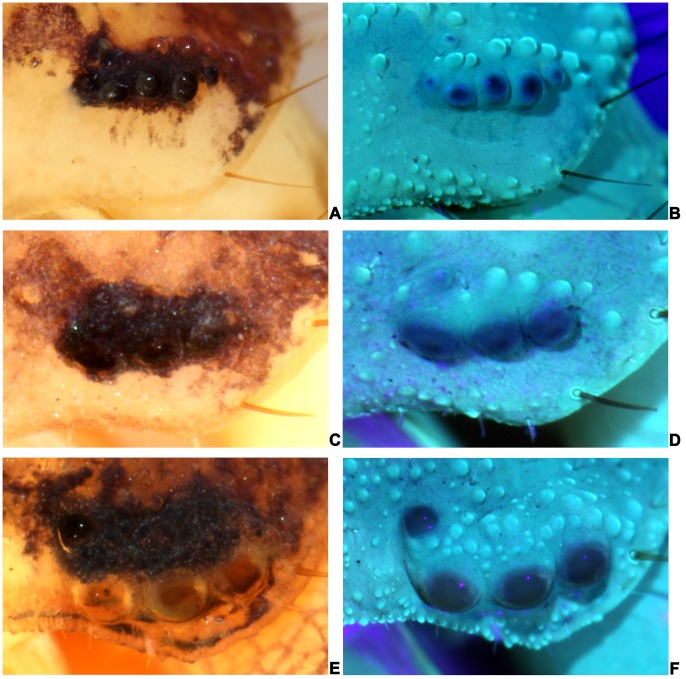
Representive specimens with exceptional number of lateral eyes. (A)&(B) A specimen with 6 lateral eyes, *M. caucasicus intermedius*, (C)&(D) A specimen lacking No. 4 lateral eye, *M. martensii martensii*, (E)&(F) A specimen lacking No. 5 lateral eye, *L. mucronatus*.

Besides, a total of 353 specimens belonging to 20 species and subspecies in 8 genera were re-examined successively to see how many buthids exhibit this number and whether they were reported properly within our collection. The statistical results were tabulated in Table 1. Selected representatives and exceptional specimens were photographed (Figure 2, Figure 3 and Figure 4). All except 5 species and subspecies previously reported with 3 or 4 pairs of lateral eyes, on re-examination had 5 pairs. The two pairs of minute lateral eyes, No. 4 and No. 5 have been overlooked.

Furthermore, we conducted reference investigations to see how many previously reported buthids with 5 pairs of lateral eyes and what and where they are. Table 2 is a summary of related publications we found, involving 63 species from 16 genera. Together with those in Table 1, this would add up to 79 species and 21 genera. Though this would account for only a small proportion in the family Buthidae and not exhaustive, these examples come from many different locations scattering Asia and Africa where contains all the buthid genera in the Old World what make it can not be easily ignored.

**Table 2:** Species and Publications with Descriptions of Five Pairs of Lateral Eyes.

Species	Location(s)	Reference(s)	Species	Location(s)	Reference(s)
*Apistobuthus susanae*	Asia: Iran	[27]	*Isometrus*(*Reddyanus*)*isadensis*	Asia: India	[20] (originally depicted as *Isometrus (Raddyanus) isadensis)*
*Apistobuthus pterygocercus*	Asia: Arabia, Oman	[27], [28]	*Isometrus*(*Reddyanus*)*acanthurus*	Asia: India	[20] (originally depicted as *Isometrus (Raddyanus) acanthurus)*
*Androctonus funestus*	Africa: North Africa	[29]	*Isometrus*(*Reddyanus*)*corbeti*	Asia: India	[20] (originally depicted as *Isometrus (Raddyanus) corbeti)*
*Androctonus australis*	Africa: North Africa	[7], [8], [30]	*Lychas gravelyi*	Asia: Burma	[20]
*Androctonus finitimus*	Asia: Pakistan, India	[20](originally depicted as *Androctonus australis finitimu*)	*Lychas nigristernis*	Asia: India	[20]
*Buthoscorpio rayalensis*	Asia: India	[31]	*Lychas biharensis*	Asia: India	[20]
*Buthoscorpio indicus*	Asia: India	[32]	*Lychas kamshetensis*	Asia: India	[20]
*Buthoscorpio sarasinorum*	Asia: Sri Lanka	[32]	*Lychas rugosus*	Asia: India	[20] (originally depicted as (*Lychas (Alterotrichus) rugosus)*
*Buthacus agarwali*	Asia: India	[33]	*Lychas hendersoni*	Asia: India	[20] (originally depicted as (*Lychas (Alterotrichus) hendersoni)*
*Buthacus arenicola maroccanus*	Africa: Marocco	[34]	*Lychas tricarinatus*	Asia: India	[20] (originally depicted as (*Lychas (Endotrichus) tricarinatus)*
*Buthacus leptochelys algerianus*	Africa: Algeria	[34]	*Lychas laevifrons*	Asia: India	[20] (originally depicted as (*Lychas (Endotrichus) laevifrons)*
*Buthacus birulai*	Africa: Algeria	[34]	*Lychas scaber*	Asia: India	[20] (originally depicted as (*Lychas (Endotrichus) scaber)*
*Buthacus mahraouii*	Africa: Morocco	[35]	*Lychas albimanus*	Asia: India	[20] (originally depicted as (*Lychas (Endotrichus) albimanus)*
*Buthiscus politus*	Asia: India	[20] (originally depicted as *Stenochirus Politus*)	*Lychas biharensis*	Asia: India	[20] (originally depicted as (*Lychas (Endotrichus) biharensis)*
*Buthiscus sarasinorum*	Asia: India	[20] (originally depicted as *Stenochirus sarasinorum*)	*Lychas kamshetensis*	Asia: India	[20] (originally depicted as (*Lychas (Endotrichus) kamshetensis)*
*Compsobuthus nematodactylus*	Asia: Oman	[36]	*Lychas aareyensis*	Asia: India	[37]
*Compsobuthus* *rugosulus*	Asia: India	[20] (originally depicted as*Compsobuthus* *acutecarinatus rugosulus*)	*Mauritanobuthus geniezi*	Africa:Mauritania	[38]
*Compsobuthus atrostriatus*	Asia: India	[20] (originally depicted as *Vachonus atrostriatus*)	*Orthochirus krishnai*	Asia: India	[20], [39]
*Hemibuthus* *crassimanus*	Asia: India	[20]	*Orthochirus pallidus*	Asia: India	[20]
*Hottentotta hottentotta*	Not mentioned	[1] (originally depicted as *Tityus hottentotta*)	*Orthochirus* *flavescens*	Asia: India	[20]
*Hottentotta saxinatans*	Asia: Oman	[40]	*Orthochirus bicolor*	Asia: India	[20]
*Hottentotta pellucidus*	Asia: Oman	[40]	*Orthochirus bastawadei*	Asia: India	[41]
*Hottentotta penjabensis*	Asia: India	[20] (originally depicted as *Buthotus alticola punjabensis*)	*Orthochirus scrobiculosus*	Asia: India	[20] (originally depicted as *Orthochirus melanurus*)
*Hottentotta flavidulus*	Asia: Afghanistan	[42]	*Odontobuthus odonturus*	Asia: India	[20] (originally depicted as *Odontobuthus doriae odonturus*)
*Hottentotta rugiscutis*	Asia: India	[20] (originally depicted as *Mesobuthus rugiscutis*)	*Parabuthus transvaalicus*	Not mentioned	[43]
*Hottentotta pachyurus*	Asia: India	[20] (originally depicted as *Mesobuthus pachyurus*)	*Parabuthus glabrimanus*	Africa: Namibia	[44]
*Isometrus* (*Isometrus*) *sankeriensis*	Asia: India	[45]	*Parabuthus calvus*	Africa: South Africa	[46]
*Isometrus* (*Reddyanus*) *brachycentrus*	Asia: India	[45]	*Parabuthus muelleri*	Africa: Namibia	[47]
*Isometrus* (*Reddyanus*) *vittatus*	Asia: India	[20] (originally depicted as *Isometrus (Raddyanus) vittatus*	*Pseudolychas pegleri*	Africa: South Africa	[48]
*Isometrus* (*Reddyanus*) *rigidulus*	Asia: India	[20] (originally depicted as *Isometrus (Raddyanus) rigidulus)*	*Vachonus rajasthanicus*	Asia: India	[20]
*Isometrus* (*Reddyanus*) *brachycentrus*	Asia: India	[20] (originally depicted as *Isometrus (Raddyanus)*	*Vachonus rajasthanicus*	Asia: India	[20]
*Isometrus* (*Isometrus*) *thurstoni*	Asia: India	[20] (originally depicted as *Isometrus (Raddyanus) thurstoni & Isometrus (Closotrichus) sankeriensis*)			

During the reexamination, we found variations in the number and location of lateral eyes. The counts of numbers of eyes ranged between 4 to 6 (Figure 3, Figure 4 and Table 1). The eye No. 4 has three possible locations, P1, P2 or P3 (Figure1. B). Most are at P1 or P2 and very few at P3 (Figure 3. Q, R, S, T) among different species.

As another result of the above work, we summarized possible reasons leading to overlooking on lateral eyes No. 4 and No. 5 in previous studies here.

1. Eyes No. 4 & 5 were smaller in size in contrast to No. 1–3 lateral eyes (only about one third to half the size of No. 1–3 eyes, Figure 2).
2. Under the stereomicroscope, the eyes No. 4 & 5 were much resembling tubercles on the carapace and almost indistinguishable in size and shape in most cases, (Figure2. B). This also could be due to the semi-transparent appearance of either the tubercles or micro-lateral eyes when under the normal white or yellow light (Figure2. B).
3. The sizes and locations of the lateral eyes were variable among different species and also among different specimens in one species. In some cases, the No. 4 was bigger than No. 5, in other cases, it was the opposite, and in most cases they were almost equivalent in size. Recognition and location was particularly difficult when the two eyes were both smaller than the average sizes.
4. Exceptions as shown in Figure 4 with insufficient sampling could be another reason contributing to this issue.

Also, we acquired a number of protocols proved to be useful in the process of reexamining the specimens, in increasing the accuracy in determining, recognizing and locating No. 4 & No. 5 lateral eyes.

1. The position of No. 4 could be variable. On the carapace (dorsal view), three types were apparent: (1) Posterior-lateral (Figure 1. B P1), similar to the positions of No. 1–3, (2) Posterior, at the posterior end of lateral eye carina composed with tubercles (if present) (Figure 1. B P2), (3) Dorsal-posterior, locating at the inner side of the line composed with lateral eye tubercles (if present) (Figure 1. B P3). Although the variation may be slight but the No. 4 could easily be confused with the many tubercles within ocular area of lateral eyes.
2. Using the Figure 1 (B, C & D) with reference of angles θ, φ is a helpful guide in determining the locations of the two lateral eyes.
3. Changing different directions of view during the examination of specimens will help in locating the No. 4 and No. 5 lateral eyes. Placing specimens well submerged in alcohol in beakers rather than in shallow alcohol of petri dishes would help in the examination as well as in the photography.
4. The lateral eyes have no fluorescence under the UV light [49], because the laterals eyes are composed with lens outside, while the tubercles are actually thickened cuticle and thus exhibit stronger fluorescence. Such characteristics can be exploited to distinguish lateral eyes from tubercles, no matter how minute these lateral eyes might be (Figure 3. H, J, L, N, V).
5. The cuticle of the scorpion is almost transparent. Each lateral eye has light sensitive dark tissue under the lens. Therefore, most researchers recorded dark “splash” around the lateral eyes, while they were not real splashes. Even if there were pigments near the lateral eyes, the darkening would be different. When specimens were anatomized, the pigments was found to be located in a thin pigment layer just beneath the cuticle, while the light sensitive dark tissues were much thicker and separated from the cuticle. The No. 5 is usually located in a separated dark “splash” or on a protruding “splash” connecting with the “splashes” around the No. 1–4.
6. Five pairs of lateral eyes are much easily seen in specimens of early instars. In a check of 27 specimens of second instar *M. martensii martensii*, no tubercle was found as big as the No. 4 or No. 5. So, this provides us an efficient and effective way to confirm this character.
7. In some specimens with light sensitive eye tissues separated from the lens for some reason, we could find some tissue scraps forming complete or half circle around the inner edges of the lens, while the pigment did not present like this around the inner side of tubercles. In such specimens this would assist in locating the eyes No. 4 and No. 5. And also, according to this phenomenon, these two lateral eyes could be well recognized by splitting the cuticle and finding the lens adhered with dark tissue from the inner side.
8. Taking photos under UV light would produce clear and sharp images of the lateral eyes which would help in situations when the lateral eyes are too small or the fluorescence is too faint to confirm by using any of the methods outlined above.
9. Lateral eyes No. 4 and No. 5 have different features providing us a method to distinguish them from each other. (1) The angle θ of No. 4 is always >90 degrees and <180 degrees, while the angle φ of No. 5<90 degrees (Figure 1. C, D; Figure 2. A, B; Figure 3). (2) Lateral eye No. 4 always looks towards posteriorly, posterior-laterally or dorsal-posteriorly, while the No. 5 always looks towards dorsally (Figure 1. B; Figure 2. A, B, C; Figure 3).

## Discussion

Difficulties in recognizing the No. 4 and No. 5 lateral eyes were first reported by Lankester & Bourne [29] who stated: “The smaller lenses are equally entitled to count as eyes with the larger. It is, however, difficult without great care and minute examination to distinguish mere tubercles of the chitinous integument from eye-lenses.” But their studies were concerned cells and tissues, and missed the attention of taxonomists. Kovařík [50] found four lateral eyes for *Butheolus* species, and pointed out that “the fourth eye” “may possibly be overlooked”. In our study, we found species *M. martensii* [9], [10], [11], [15], *M. caucasicus* [11], [15], [16], [17], [51] and *M. eupeus* with their subspecies [11], [12], [13], [14], [15], [51] were studied numerous of times. Though there was no lack of good descriptions with drawings or high-tech photography among these studies, e.g. Qi, et al. [10], Sun & Zhu [16], Mirshamsi, et al. [14], Sun & Sun [11], these “extra” lateral eyes were also not mentioned. Even the scanning electron microscopy were used in morphological studies, the same case still occurs, for example, Figure 2 of Lourenço & Leguin [26] illustrated a “Five-eye” model, but unfortunately, the No. 4 and 5 lateral eyes were not recognized. Therefore, the “overlooking” of the No. 4 and 5 lateral eyes should be a common gap.

Schliwa & Fleissner [7] measured lateral eyes of *Androctonus australis* and reported “approximately 0.3 mm” for No. 1–3 and “ranging from 0.1–0.2 mm” for No. 4 & 5. Kishida [9] made a good re-description for *M. martensii* with five lateral eyes. What need to be highlighted here is Tikader and Bastawade’s work [20]. In their book “Fauna of India, Scorpions”, all Buthidae species and subspecies (43 Species and subspecies) except those in the genus *Charmus* were precisely depicted and illustrated with five pairs of lateral eyes. Besides, Finnegan (1932), Fleissner (1974), Schliwa & Fleissner (1980), Qi & Lourenço (2007), Lowe (2010), Javed, et al. (2010) provided useful depictions, figures or photos in their publications, other taxonomists involved in the Table 2 “References” provided dozens of reports for the model. These researches greatly support the work reported here and further made it possible to draw a general conclusion.

As a result, we confirm that all the Chinese scorpion species of buthids examined here, whether reported in new (most recent in 2011) or old (latest backdated to 1778) publications, have five pairs of lateral eyes. Together with related descriptions published by Tikader and Bastawade [20], we are convinced that the “Five-eye” model is appropriate to incorporate in descriptions of the genus *Mesobuthus*. Combined with more other related data collected in Table1 & Table 2, this model is likely to be generic character of *Hottentotta*, *Lychas*, *Isometrus*, *Buthacus*, and *Orthochirus*. And also, we could further conclude that there would be a possibility too that this model could be a general feature in more other buthid genera with much more species that could be found to have five pairs of lateral eyes.

It should not only be a coincidence that the majority of Buthidae species from India and China falls within the “Five-eye” model. Moreover, with examples from Asia and Africa including those from type genus, we have reasons to believe that a general significance to this model in the Old World buthid genera is possible and further in the whole Buthidae family, as majority of buthid genera distribute in the Old World, mainly Asia and Africa [52], [53]. Although, it can now be confirmed that the “Five-eye” model is much more common among buthid species and genera than what is known before. In addition, this study has managed to prove a general trend of oversight in recognizing the presence of lateral eyes No. 4 and 5.

We propose four main factors which contributed to this error:

1. Difficulties faced during examining of specimens;
2. Traditional and insufficient examining methods;
3. Lack of development of the lateral eyes in some of the specimens;
4. Insufficient number of samples examined during the study.

As reported in the results, most of the difficulties could be overcome by the use of UV light as well as new examining protocols. Variation in the number of lateral eyes among different specimens of the same species would be common in spite it is very small in proportion (Table 1). Therefore, this error might occur when small samples are used in the studies.

With this general trend of oversight, we believe we are at the tip of major discoveries amidst many arising questions in search of answers: What status of eye numbers are there in the other genera and species in Buthidae? How many genera and species have this “Five-eye” model are there? Are there other models with four pairs of lateral eyes (as in the genus *Charmus*) or less, where are they and which eye model holds the majority?

Clarifying the use of character ‘number of lateral eyes’ is important not only in taxonomic studies but also in other research areas, such as evolution and behavior. As there are nearly 1000 species and 90 genera in Buthidae, most of the published species need to be rechecked are suggested to be rechecked here. This would need concerted effort from all concerned in order for us to be able to draw the conclusion and appreciate the contributing importance of this character in the taxonomy of scorpions.
