# Correction: True Lateral Eye Numbers for Extant Buthids: A New Discovery on an Old Character

**DOI:** 10.1371/annotation/434bd393-ae8c-47fe-a2a9-f80ef71183f4

**Published:** 2013-09-20

**Authors:** Xiao Feng Yang, Yusoff Norma-Rashid, Wilson R. Lourenço, Ming Sheng Zhu

The initials for the first and fourth authors are incorrect. The correct initials are Yang XF and Zhu MS. The correct citation is: Yang XF, Norma-Rashid Y, Lourenço WR, Zhu MS (2013) True Lateral Eye Numbers for Extant Buthids: A New Discovery on an Old Character. PLoS ONE 8(1): e55125. doi:10.1371/journal.pone.0055125 

